# Starvation at the larval stage increases the vector competence of *Aedes aegypti* females for Zika virus

**DOI:** 10.1371/journal.pntd.0010003

**Published:** 2021-11-29

**Authors:** Christie S. Herd, DeAna G. Grant, Jingyi Lin, Alexander W. E. Franz

**Affiliations:** 1 Dept. of Veterinary Pathobiology, University of Missouri, Columbia, Missouri, United States of America; 2 University of Missouri Electron Microscopy Facility, Columbia, Missouri, United States of America; University of Wisconsin Madison, UNITED STATES

## Abstract

*Aedes aegypti* is the primary vector of Zika virus (ZIKV), a flavivirus which typically presents itself as febrile-like symptoms in humans but can also cause neurological and pregnancy complications. The transmission cycle of mosquito-borne arboviruses such as ZIKV requires that various key tissues in the female mosquito get productively infected with the virus before the mosquito can transmit the virus to another vertebrate host. Following ingestion of a viremic blood-meal from a vertebrate, ZIKV initially infects the midgut epithelium before exiting the midgut after blood-meal digestion to disseminate to secondary tissues including the salivary glands. Here we investigated whether smaller *Ae*. *aegypti* females resulting from food deprivation as larvae exhibited an altered vector competence for blood-meal acquired ZIKV relative to larger mosquitoes. Midguts from small ‘Starve’ and large ‘Control’ *Ae*. *aegypti* were dissected to visualize by transmission electron microscopy (TEM) the midgut basal lamina (BL) as physical evidence for the midgut escape barrier showing Starve mosquitoes with a significantly thinner midgut BL than Control mosquitoes at two timepoints. ZIKV replication was inhibited in Starve mosquitoes following intrathoracic injection of virus, however, Starve mosquitoes exhibited a significantly higher midgut escape and population dissemination rate at 9 days post-infection (dpi) via blood-meal, with more virus present in saliva and head tissue than Control by 10 dpi and 14 dpi, respectively. These results indicate that *Ae*. *aegypti* developing under stressful conditions potentially exhibit higher midgut infection and dissemination rates for ZIKV as adults, Thus, variation in food intake as larvae is potentially a source for variable vector competence levels of the emerged adults for the virus.

## Author summary

When mosquitoes are reared in a laboratory they are typically provided with ample nutrients as larvae so adults can grow to an optimal size; this ensures adults are robust for reproducible experiments. However, in the field not all larvae may have access to equal amounts of food. Studies including ours have shown that by restricting food as larvae, smaller adults can be produced, which can have an altered ability to be infected with and transmit arthropod-borne viruses. Zika virus is ingested into a female mosquito midgut when a blood-meal is acquired from an infected vertebrate host; the virus must infect midgut cells and escape this tissue to secondary tissues via the basal lamina, which surrounds the midgut. Viruses can then infect other organs including the salivary glands, for further transmission. In this study we focus on the impact limited nutrition as a larva has on the adult’s transmission potential for Zika virus.

## Introduction

*Aedes aegypti* mosquitoes are the primary vectors of Zika virus (ZIKV; *Flaviviridae*, *Flavivirus*) [1], which typically causes febrile illness when transmitted to humans, but can also cause serious health conditions such as neurological Guillian Barré syndrome, and pregnancy complications including stillbirth and microcephaly in unborn children [2–4][reviewed in [5]. Prior to 2015, ZIKV was endemic in African and Asian countries, however, a large outbreak in the Americas led to a surge in imported travel cases in countries previously absent of the virus [6]. Consequently, following travel-imported cases, autochthonous transmission of ZIKV has meanwhile occurred in 87 countries and territories across Africa, the Americas, South-East Asia and the Western Pacific [7]. Although ZIKV cases have declined in recent years, the threat of renewed outbreaks in the future is continuing, justifying the need to study the factors influencing mosquito vector competence for the virus.

Vector competence studies typically use mosquitoes reared under standard laboratory conditions, with ample food provision and low rearing density to produce healthy adults. However, mosquito larvae grown up in bulk under optimal rearing conditions may still ingest variable amounts of food at the individual level. Once *Ae*. *aegypti* eggs hatch, larvae require food to moult through four instars, transitioning into non-feeding pupae that will eclose into winged adults [8]. The fourth instar larva must reach a critical mass, upon which production of juvenile hormone stops as the larva commits to pupation. The size the larva has reached at this stage eventually determines the adult body mass [9]. Once a larva moults into a fourth instar, if feeding is then suspended, so is the development into a pupa. Interestingly, these fourth instar larvae have been shown to tolerate starvation conditions for up to two weeks, resuming pupation if fed again [10].

Mosquito-borne arthropod-borne viruses (arboviruses) are horizontally transmitted from a viremic vertebrate host to female mosquitoes when they acquire a blood-meal to develop their eggs. Arboviruses ingested along with a blood-meal encounter several physical barriers in the mosquito including initial midgut epithelial cell infection, midgut escape, salivary gland infection and saliva transmission [11]. The virions that successfully establish an infection of the midgut epithelium must overcome the midgut escape barrier for dissemination to secondary organs such as the salivary glands, to ensure further transmission. The midgut escape barrier has been shown to be an important barrier in the systemic arbovirus infection of mosquitoes, imposing a genetic bottleneck on RNA viruses including ZIKV [12,13]. The midgut is surrounded by the BL, an interconnected grid-like network containing collagen and laminin with an average mesh size of 30nm [14]. Following ingestion of a blood-meal, the midgut dramatically distends leading to a disruption of individual strands of the BL and an increase of the BL pore size exclusion limit. This then allows virions of >30nm in diameter to traverse this barrier [15]. Thus, the midgut BL is a dynamic structure that undergoes profound changes during blood-meal digestion. ZIKV has been shown to disseminate from the *Ae*. *aegypti* midgut as early as 72 hours post-acquisition of an artificial virus containing blood-meal [16].

The infection cycle of arboviruses is well documented in large, well-fed mosquitoes, however, there is evidence that providing sub-optimal nutrients during larval development can result in small size adults with altered gene expression profiles and vector competence for arboviruses, in comparison to those mosquitoes reared under optimal conditions [17–21]. Inducing various stressors including nutrient deprivation, elevated temperatures, and treatment with insecticide at the larval stage increased the susceptibility of adult *Ae*. *aegypti* to alphavirus Sindbis virus (SINV; *Togaviridae*, *Alphavirus*) infection and dissemination relative to the controls [17,18]. Larval starvation was associated with significant downregulation of endogenous genes such as HSP70, HSP83, cecropin, defensin, transferrin, and CYP6Z6 suggesting that mosquito larvae may reduce their investment in defence and immunity when confronted with starvation [17]. The limited resources in the starved larvae seem to be made available for survival, growth, and development at the cost of resistance to infections (including those with arboviruses), which may also transiently affect the emerging adults.

As well as perturbing physiological processes, nutrient deprivation as larvae can also affect the efficacy of physical barriers such as the midgut escape barrier. Small *Ae*. *triseriatus* transmitted the orthobunyavirus LaCrosse virus (LACV; *Peribunyaviridae*, *Orthobunyavirus*) to mice at a higher rate (82%) than large, optimally reared mosquitoes (52%) [19]. While 100% of midguts were infected in both small and large mosquitoes, LACV dissemination rate was higher in small mosquitoes (50%) compared to large ones (16%). This shows that despite efficient midgut infection, the midgut escape barrier is an important barrier which may have been weakened in the nutritionally deprived *Ae*. *triseriatus*. Indeed, transmission electron microscopy (TEM) showed the mean BL width of large *Ae*. *triseriatus* mosquitoes was 0.23μM, compared to 0.14μM in small mosquitoes [22]. The BL thickness of three *Ae*. *albopictus* strains differed in correlation with the dissemination efficiency of dengue-1 virus (DENV1; *Flaviviridae*, *Flavivirus*) from the midgut. The OAHU strain exhibited 90% midgut escape of DENV1, followed by HOUS (62%) and NORL (46%); the mean BL thickness was 0.091μM, 0.192μM and 0.175μM, respectively, which shows the strain with the highest midgut escape rate had the thinnest BL [21]. On the other hand, there was no difference in the BL thickness of NORL mosquitoes with and without disseminated DENV1 infections, suggesting the BL thickness had no impact on virus dissemination [21].

Here, we investigate what effect the nutrient deprivation at the larval stage has on the vector competence of adult *Ae*. *aegypti* for ZIKV when orally acquired along with a blood-meal. Small nutritionally deprived (Starve) and large (Control) *Ae*. *aegypti* were compared in terms of midgut, carcass, head tissue and saliva infection with ZIKV. The midgut BL was imaged using transmission electron microscopy (TEM) at various timepoints and the width of the BL was measured in Starve and Control mosquitoes. We show that smaller Starve *Ae*. *aegypti* have an increased transmission potential for ZIKV and a thinner midgut BL relative to Control mosquitoes.

## Methods

### Mosquito rearing to produce small and large *Ae*. *aegypti*

Eggs of the *Aedes aegypti* Higgs White Eye (HWE) [23] strain were hatched in water supplied with 0.03g of tropical fish food (Tetra, Melle, Germany). Larvae were reared at a density of 200 per shoe-box size container filled with 800ml of distilled water and fed different amounts of food to produce small and large adults as follows. Control (large) mosquitoes were produced by supplying 0.15g of ground fish food as outlined in **S1 Table**, ensuring there were no periods without food available. Small (Starve) mosquitoes were produced by providing 0.08g food with a two-day starvation period as L1 larvae. To adjust for delayed pupation times, starved mosquitoes were hatched two days earlier than control mosquitoes (**S1 Table**). Upon eclosion, wings were removed from a sub-set of mosquitoes from each treatment and mounted to a microscope slide with double-sided Scotch tape. Wings were imaged using a Leica EZ4 W stereo microscope (Leica Camera, Wetzlar, Germany) with in-built camera and captured images were analysed using ImageJ. Adults were kept in cardboard cups with netting and supplied with water cups and raisins. Care was taken to ensure adults were the same age in both groups when sampled for various timepoints. All life stages were reared in an insectary at 28°C and 80% humidity under a 12h light/dark cycle.

### ZIKV propagation and infection of mosquitoes via virus-containing blood-meals

Adult female mosquitoes were challenged with a ZIKV-containing blood-meal at 5 days post-eclosion. ZIKV I-44 strain (Genbank: KX856011) was used for challenges, isolated from mosquitoes from Mexico in 2016 [1,24]. Prior to its use in vector competence studies, the virus had been serially passaged four times in Vero cells. ZIKV was added to 90% confluent Vero cells (ATCC: CCL-81) at multiplicity of infection (MOI) 0.01 for 96–120 hours or until 70% cytopathic effect (CPE) was observed. Vero cells were grown in Dulbecco’s Modified Eagle Medium (DMEM) supplemented with 7% fetal bovine serum (FBS). Virus-containing supernatant was harvested and used immediately for artificial feedings in a 1:1 ratio with defibrinated sheep blood (Colorado Serum Company, Denver, CO, USA) supplemented with 10mM ATP to stimulate feeding. For each mosquito carton, the blood-meal was supplied through a parafilm (Thermo Fisher Scientific, Waltham, MA, USA) membrane stretched over a glass feeder, with the glass feeder heated by a water-jacket to 37°C. Mosquitoes were provided the infectious blood-meal for up to 1 hour and anaesthetised on ice for selection of engorged females, which were then kept in cardboard cartons in a humidified chamber at 28°C and 80% humidity until sampled.

### ZIKV plaque assay on individual mosquito tissues

For each experiment, tissues were dissected at various timepoints following a ZIKV-containing blood-meal and immediately frozen in dry ice. To assess the midgut escape barrier, midguts and carcasses were sampled at 5, 7, 8, 9, and 11 dpi (dpi refers to days post-infection with a ZIKV containing blood-meal) in one experiment and 3, 5, 7 and 9 dpi in a second experiment. To analyse viral dissemination from the midgut to secondary tissues, heads and carcasses were dissected at 10 and 14 dpi. All samples were then stored at -80°C until homogenized in 0.5mL of DMEM (supplemented with 7% FBS and 5% HEPES) using a hand-held homogenizer followed by filtration through 0.22μM Supor Membrane syringe filters (Pall Life Sciences, East Hills, NY, USA). Filtered samples were 10-fold diluted in 96-well plates and 150μl of each sample from each well of the dilution series was then transferred to infect confluent Vero cells in 24-well plate formats for 1 hour at 37°C and under 5% CO_2_ supplement, while rocking every 15 minutes. A 1% agarose and nutrient mixture consisting of 10% M199 (10x), 7% FBS, 0.5% MEM non-essential amino acids (100x), 0.5% MEM vitamin solution (100x), and 0.003% sodium bicarbonate (Gibco, ThermoFisher, Waltham, MA, USA) was then overlaid on cells. Plates were incubated at 37°C and under 5% CO_2_ supplement for 5 days, then fixed with 10% formalin for 4 hours. Agarose was removed using a spatula and plaques/cells stained using 0.2% crystal violet solution, then counted to calculate viral titre (defined as plaque forming units (PFU)/mL).

### Immunofluorescence assays (IFA) to detect ZIKV antigen in midguts

Midguts were dissected at 5, 7, 8, 9, and 11 days following challenge with a ZIKV-containing blood-meal and fixed in 4% paraformaldehyde diluted in phosphate buffer solution (PBS; Gibco, ThermoFisher, Waltham, MA, USA) at 4°C for at least 30 minutes. Midguts were then permeabilized in PBS-T (PBS with 1% BSA and 0.2% Triton-X-100) for 1 hour at room temperature (RT) followed by incubation at 4°C overnight with the primary (monoclonal) antibody Anti-Flavivirus Group Antigen D1-4G2-4-15 (ATCC: VR-1852) diluted 1:500 in PBS-T. Samples were then washed three times with PBS-T with each wash step lasting 5 minutes. The secondary (monoclonal) antibody, goat anti-mouse IgG labelled with Alexa Fluor 594 (Abcam: ab150120) was added at a 1:500 dilution in PBS-T for 1 hour at RT in the dark, along with Alexa Fluor Phalloidin 488 (Invitrogen, Carlsbad, CA, USA) at a 1:1000 dilution. Cell nuclei were stained with DAPI (Invitrogen) at 1μg/mL for 10 minutes at RT. Midguts were washed three times with PBS-T again and mounted onto six-well slides using Fluoromount G mounting medium (Electron Microscopy Sciences, Hatfield, PA, USA). Samples were imaged using an inverted spectral confocal microscope (TCP SP8 MP, Leica Microsystems, Wetzlar, Germany) at the Molecular Cytology Core of the University of Missouri.

### Transmission electron and scanning transmission electron microscopy (TEM & STEM) on midgut samples

Midguts were collected from mosquitoes at 3 and 5 days post-ingestion of a ZIKV-containing blood-meal and fixed in a solution containing 2% paraformaldehyde, 2% glutaraldehyde and 100mM sodium cacodylate buffer, pH 7.35 (Sigma Aldrich, St. Louis, MO, USA) for at least 30 minutes at 4°C. Sample processing for TEM including embedding and ultrathin-sectioning were performed at the Electron Microscopy Core at the University of Missouri. Samples were embedded in HistoGel (Thermo Scientific, Kalamazoo, MI, USA) and rinsed in 100mM sodium cacodylate buffer containing 130mM sucrose. A second fixation was performed in a Pelco Biowave (Ted Pella, Redding, CA, USA) using 100mM sodium cacodylate buffer supplemented with 1% osmium tetroxide. Samples were fixed for 1 hour at 4°C, then *en bloc* stained overnight at 4°C with 1% aqueous uranyl acetate. A graded dehydration series (100 Watts for 40 sec per exchange) was performed from ethanol to acetone. Dehydrated specimens were infiltrated with EPON resin (at 250 Watts for 3 minutes) and polymerised at 60°C overnight. Embedded sections were ultrathin-sectioned (85nm) using an ultra-microtome (Ultracut UCT, EM UC7, Leica Microsystems, Wetzlar, Germany) containing a diamond knife (Diatome, Hatfield, PA, USA). TEM images were captured using a JEOL JEM 1400 transmission electron microscope connected to a Gatan Ultrascan 1000 CCD camera (Gatan, Pleasanton, CA, USA). STEM images were generated using a ThermoFisher Tecnai F30 Twin 300kV TEM/STEM operated at 200kV in high angle annular dark field (HAADF) image mode.

### Intrathoracic injection of ZIKV

Five-day-old *Ae*. *aegypti* females were anaesthetised on ice and intrathoracically injected as described before [25] with ZIKV at a titre of 1x10^6^ PFU/mL using a pulled capillary tube attached to a Nanoject II injector (Drummond Scientific Company, Broomall, PA, USA). Following intrathoracic injection of each female mosquito with 140 PFU of ZIKV, mosquitoes were maintained at 28°C and 80% humidity until whole bodies were sampled for plaque assay at 10 dpi.

### Saliva collection and detection of ZIKV

Ten days following provision of a ZIKV-containing blood-meal, forced salivation was performed on female mosquitoes. Mosquitoes were deprived of sugar the night before and then saliva collected the next day. Female mosquitoes were cold-anesthetized to remove wings and legs, then proboscises were inserted into 1mm glass capillary tubes filled with 5μl Cargille Type B immersion oil (Cargille labs, NJ, USA) for saliva collection over 45 minutes. The end of each capillary tube was then placed into an Eppendorf tube filled with 200μl of DMEM (supplemented with 7% FBS) and centrifuged at 3,000x*g* for 15 minutes to elute the saliva. Samples were stored at -80°C until processed. To sterile-filter saliva samples, another 300μl of DMEM (7% FBS) was added and the sample was filtered through a 0.2μm syringe filter (Pall Life Sciences, East Hills, NY, USA). Vero cells were plated out into 24-well plates and 180μl of saliva samples were inoculated in each well for one hour at 37°C while rocking every 15 minutes. Each well was supplemented with 1ml of DMEM (7% FBS) and the presence of cytopathic effects (CPE) indicating productive infection of ZIKV was monitored on a daily basis for 8 days. At least six non-infected control wells were included as a comparison for non-virus induced CPE.

### Statistical analysis

All graphics were created in R Studio (RStudio Inc, Boston, Massachusetts, USA) using the ‘ggplot2’ package. Statistical analysis was performed in R Studio. Normality was assessed using the Shapiro-Wilk test. Significance of BL width between midguts was assessed using a Kruskal Wallis test (non-parametric), followed by a post-hoc Dunn Test using the ‘FSA’ package. Compact letter display of significance was calculated using the ‘rcompanion’ package. Parametric (T-test) or non-parametric (Mann-Whitney U-test) analysis was selected depending on normal and non-normal distribution of data, respectively. Viral infection intensity in individual samples was analysed using the Mann-Whitney U-test. Fisher’s Exact Test was used for statistical analysis of viral prevalence in mosquito samples.

## Results

### Larval starvation led to the development of smaller adult mosquitoes

Once female adult Control and Starve mosquitoes had eclosed, their wings were dissected and measured as a proxy for body mass [26]. The starvation feeding regimen consistently produced Starve mosquitoes with significantly smaller wings relative to Control mosquitoes over three independent replicates (*p* ≤ 0.0001, *p* = 0.0005; Mann-Whitney U-test) (**S1 Table and S1 Fig**). The mean wing length of Starve wings was less than Control at all timepoints sampled (**Table 1**).

**Table 1:** Mean wing lengths (mm) ± standard deviation (SD) of adult *Ae*. *aegypti* (HWE) reared under Control and Starve diet regimen in three replicates.

Wing length (mm)			
	Replicate		
Diet	1	2	3
Control	2.93 ± 0.10 (21)**+**	3.13 ± 0.11 (14)	2.97 ± 0.06 (9)
Starve	2.77 ± 0.11 (22)*	2.65 ± 0.10 (21)*	2.78 ± 0.11 (9)*

*: significantly different than Control

Wing length (mm) = mean ± SD

**+**: 21 = number of wings measured

### Starve females possessed a significantly thinner midgut BL than Control mosquitoes

**Fig 1:**
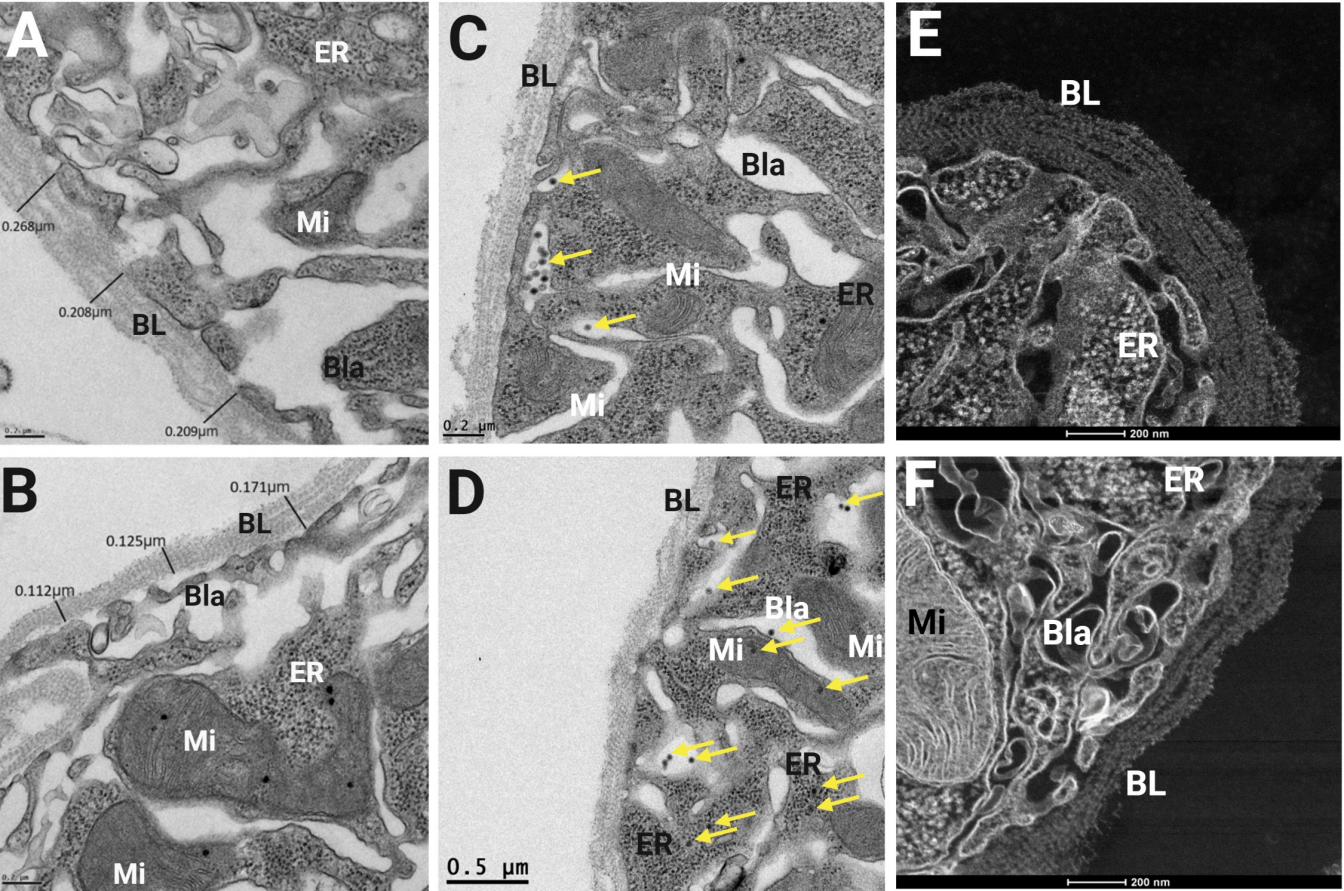
Ultrastructural (TEM) visualization of the midgut BL in Starve and Control *Ae*. *aegypti* (HWE). Midguts of Starve and Control *Ae*. *aegypti* were dissected at 3 and 5 days post-infection (dpi) via ingestion of a ZIKV-containing blood-meal and visualized by transmission electron microscopy (TEM). TEM images of (**A**) Control and (**B**) Starve midguts are shown with BL width measurements at 5 dpi. (**C**) Multiple virions are accumulating in the basal labyrinth of a Starve midgut at 3 dpi (yellow arrows). (**D**) ZIK virions are shown in a Control midgut both in the basal labyrinth and in the endoplasmic reticulum at 5 dpi. (**E**) Scanning transmission electron microscopy (STEM) images showing a higher number of individual BL strands of a Control midgut at 5 dpi when compared to the (**F**) BL of a Starve midgut. BL = basal lamina, Bla = basal labyrinth, ER = endoplasmic reticulum, Mi = mitochondria. TEM images were captured using a JEOL JEM 1400 transmission electron microscope and STEM images were captured using a ThermoFisher Tecnai F30 Twin 300kV transmission electron microscope/scanning transmission electron microscope.

Midguts from Starve and Control mosquitoes were dissected and their BL was visualized using TEM at 3 and 5 days post-ingestion of a ZIKV-containing blood-meal. These timepoints were chosen as ZIKV has been shown previously to start to disseminate from the midgut of *Ae*. *aegypti* from 3 dpi onwards [16]. Examples of BL measurements taken from Control and Starve midguts at 5 dpi are shown in **Fig 1A** and **1B**, respectively. *De novo* synthesized Zika (ZIK) virions were clearly visible at 3–5 dpi, accumulating in the basal labyrinth in proximity to the BL (Starve midgut, **Fig 1C**), and also in the endoplasmic reticulum (Control midgut, **Fig 1D**). Scanning Transmission Electron Microscopy (STEM) images show higher detail of a Control midgut with densely arranged BL strands (**Fig 1E**), compared to a section of a Starve midgut with a thinner BL composed of fewer strands (**Fig 1F**).

**Fig 2:**
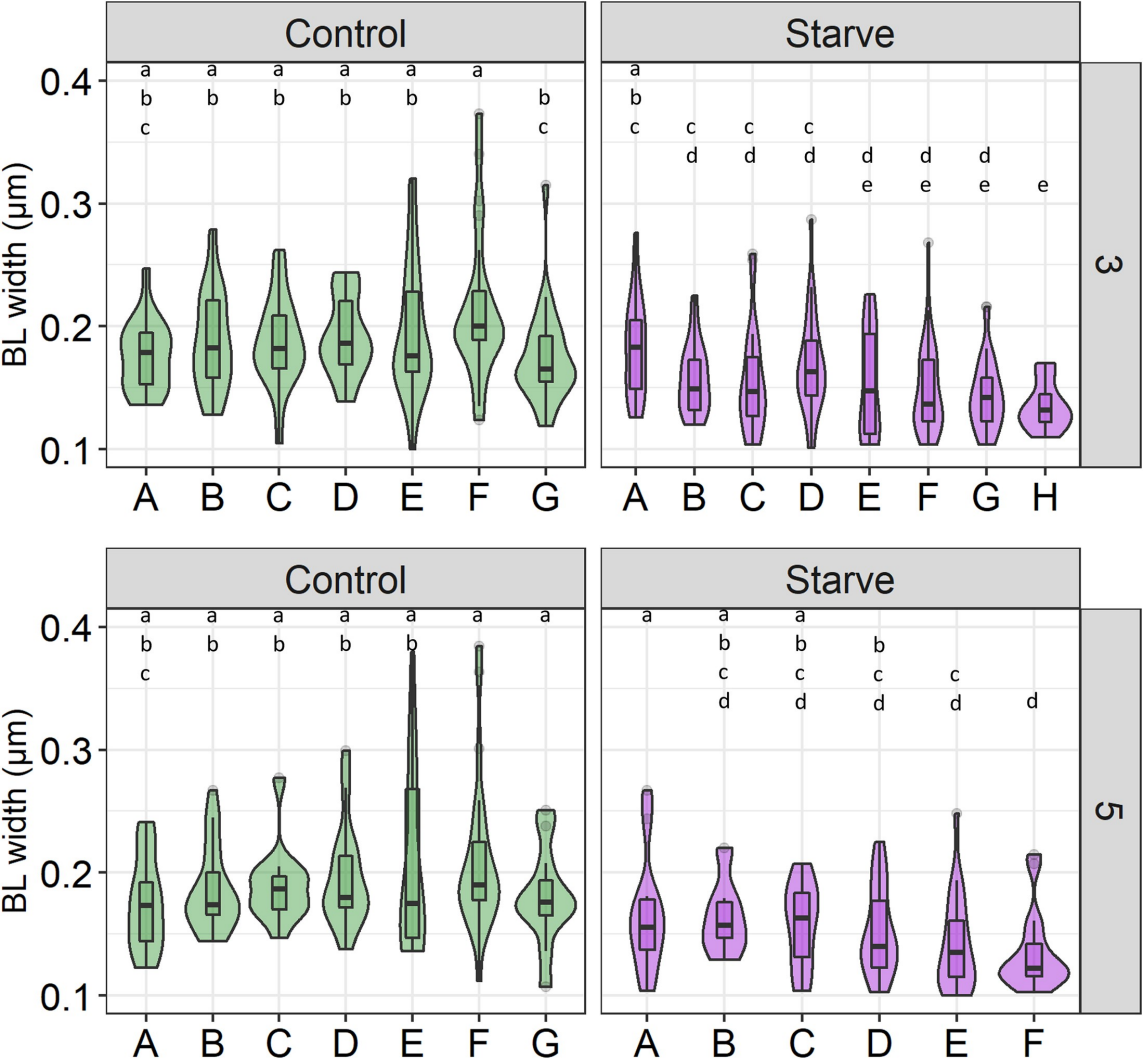
Distribution of midgut basal lamina (BL) width in individual midguts of Control and Starve *Ae*. *aegypti* HWE. Five day-old *Ae*. *aegypti* HWE Control and Starve mosquitoes were fed a ZIKV-containing blood-meal and the ultrastructure of the midgut BL was visualized at 3 and 5 days post-infection (dpi). TEM images were obtained from 6–8 Control and Starve midguts per timepoint (Midguts A-H). ImageJ was used to take three measurements of the BL width per image. Each BL measurement was plotted in the violin plot representing the distribution of BL width per midgut at 3 dpi and 5 dpi. The boxplot within the violin plot represents the median, upper and lower quartiles, and extremities. Kruskal Wallis and post-hoc Dunn Test were used for statistical analysis. Significance is shown by different letters (*p* < 0.05).

The distribution of BL width measurements from individual Control and Starve midguts showed significant differences between midguts at each timepoint as represented by different letters (**Fig 2** and **Table 2**). Mean values for BL width ranged from 0.119μm +/- 0.032 SD (Starve, 3 dpi) to 0.304μm +/- 0.185 SD (Control, 5 dpi) (**Table 2**). Within treatment groups, BL width was similar between most analysed midguts at 3 and 5 dpi. However, between groups at 3 dpi, BL of Starve midguts B-H were significantly thinner than those of Control midguts B-F (**Fig 2**). Similarly, at 5 dpi, the BL of Starve midguts D-F were significantly thinner than the BL of Control midguts B-G, with the BL of Starve midgut C being significantly thinner than BL of Control midguts F and G (**Fig 2**). None of the midgut BL in Control mosquitoes were significantly thinner than in Starve mosquitoes.

**Table 2:** Mean basal lamina width (μm) ± standard deviation (SD) in midguts of adult *Ae*. *aegypti* reared under Control and Starve diet regimens at 3 and 5 days post-infection with orally acquired ZIKV I-44.

Basal lamina width (μm)							
Timepoint							
3 DPI				5 DPI			
Control		Starve		Control		Starve	
MG	BL width	MG	BL width	MG	BL width	MG	BL width
A	0.176 ± 0.029 (18)*	A	0.142 ± 0.039 (36)	A	0.175 ± 0.037 (18)	A	0.153 ± 0.038 (12)
B	0.188 ± 0.040 (30)	B	0.158 ± 0.028 (21)	B	0.186 ± 0.034 (21)	B	0.135 ± 0.039 (24)
C	0.187 ± 0.036 (21)	C	0.119 ± 0.032 (24)	C	0.190 ± 0.032 (12)	C	0.148 ± 0.038 (27)
D	0.192 ± 0.032 (18)	D	0.161 ± 0.044 (33)	D	0.208 ± 0.061 (27)	D	0.139 ± 0.032 (36)
E	0.230 ± 0.082 (27)	E	0.134 ± 0.053 (21)	E	0.304 ± 0.185 (18)	E	0.165 ± 0.032 (6)
F	0.175 ± 0.038 (27)	F	0.155 ± 0.043 (21)	F	0.197 ± 0.043 (15)	F	0.133 ± 0.033 (18)
G	0.196 ± 0.053 (27)	G	0.178 ± 0.044 (18)	G	0.203 ± 0.073 (21)		
		H	0.166 ± 0.048 (12)				

MG = Midgut

*: 18 = number of measurements taken

Basal lamina width (μm) = mean ± SD

### Midgut infection dynamics of ZIKV in Control and Starve mosquitoes differed between experiments

**Fig 3:**
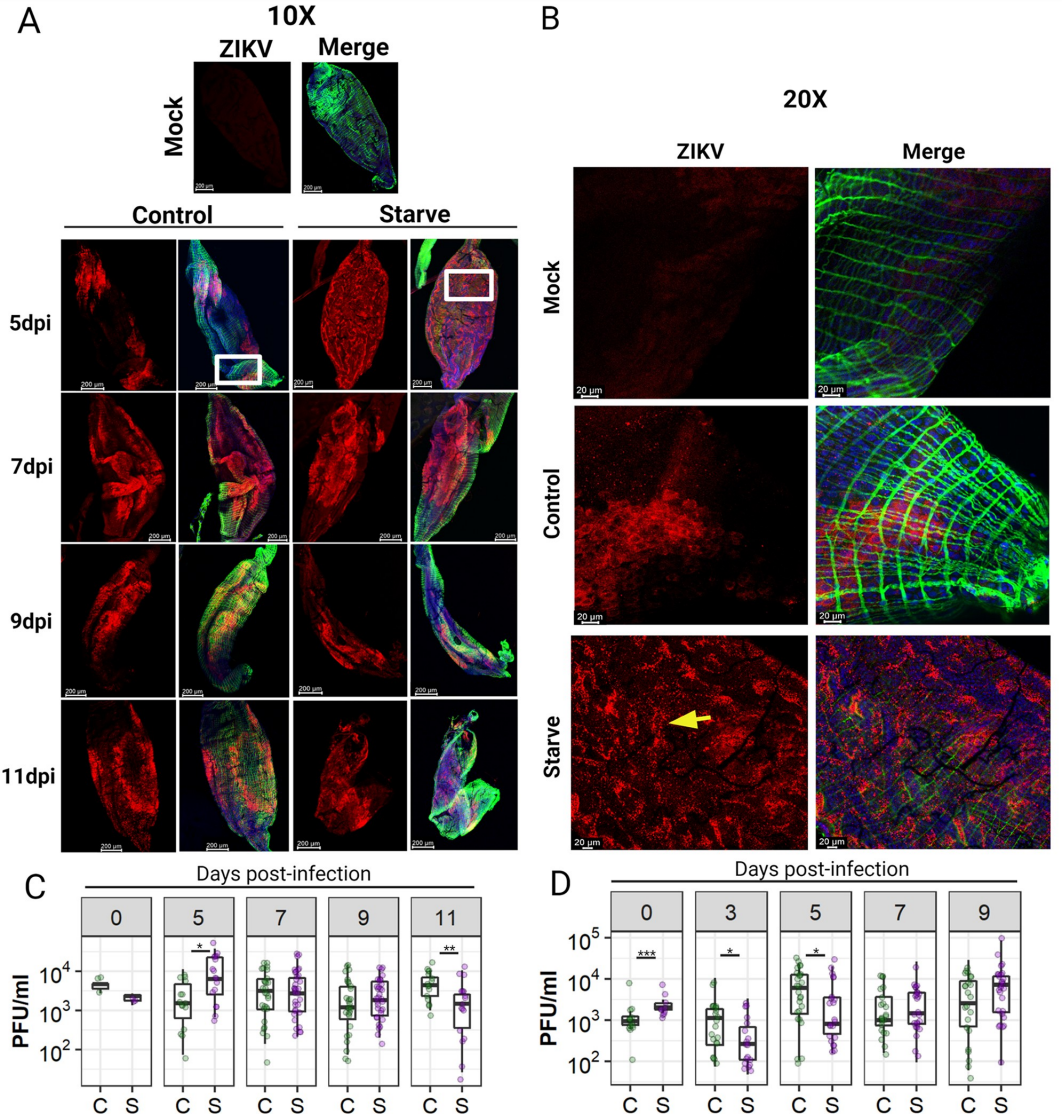
Intensity of ZIKV I-44 midgut infection in Control and Starve *Ae*. *aegypti* HWE following oral ingestion of virus. (**A**, **B**) Midguts were dissected at 5, 7, 8, 9, and 11 days post-infection (dpi) via a ZIKV containing blood-meal. ZIKV titre in the blood-meal for all challenges was 1x10^6^ PFU/mL. Fixed midguts were incubated with the flavivirus-specific 4G2 primary mouse monoclonal antibody and secondary anti-mouse Alexa Fluor (AF) 594 labeled monoclonal antibody (red). Actin filaments were stained using Alexa Fluor (AF) Phalloidin 488 (green); nuclei were stained using DAPI (blue). Mock samples show non-infected midguts which underwent the same staining procedure as the infected midguts. (**A**) Midguts were imaged at 10x magnification to show overall midgut infection pattern using an inverted spectral confocal microscope (TCP SP8 MP, Leica Microsystems). (**B**) 20x magnified images showing detailed infection foci of 5 dpi midguts (area within white square at 10x magnification). Yellow arrow indicates specific punctate infection foci in Starve midgut compared to zonal infection in the Control midgut. At least 6 midguts were visualized per group/timepoint in two independent experiments. (**C**, **D**) Midguts were dissected over a time-course following oral challenge of mosquitoes with ZIKV-containing blood-meals and virus quantified by plaque assay. (**C**) Experiment 1: ZIKV titre in midguts at 0 (immediately after blood-meal ingestion), 5, 7, 9, and 11 dpi. (**D**) Experiment 2: ZIKV titre in midguts at 0, 3, 5, 7, and 9 dpi. Boxplots show median, upper, and lower quartiles and data extremities. Dots represent individual midguts. n = 20–39 midguts per timepoint. Statistics were based on Mann-Whitney U-test; * = *p* < 0.05, ** = *p* < 0.01, *** = *p* < 0.001. C = Control, S = Starve.

ZIKV was quantified by plaque assay in Control and Starve midguts over a time-course following challenge with ZIKV-containing blood-meals in two independent experiments. In both experiments, the ZIKV concentration in the provided blood-meal was 1.7x10^6^ PFU/mL. In the first experiment, Control and Starve mosquitoes ingested on average 4,0 and 2,1 PFU/mL of virus respectively, with no significant difference *(p* = 0.08, T-test). Midgut infection rates between Control and Starve mosquitoes remained relatively constant throughout the first experiment, reaching 75–89% infection in both groups (**Table 3**). Patterns of ZIKV infection were generally similar in midguts of Starve and Control mosquitoes between 5–11 dpi based on *in situ* detection of viral antigen (**Fig 3A**), although in 2/6 Starve midguts, we observed a widespread punctate ZIKV infection pattern at 5 dpi (**Fig 3B**).

**Table 3:** Median ZIKV titre (plaque forming units per mL), infection rates, and midgut escape rates in Control and Starve midguts and carcasses over a time-course in two independent experiments.

			Experiment 1				Experiment 2			
			5 DPI	7 DPI	9 DPI	11 DPI	3 DPI	5 DPI	7 DPI	9 DPI
Midgut	Median titre (PFU/mL)	Control	1,533	3,117	1,200	4,333*	1,133*	6,000*	1,000	2,600
		Starve	6,300*	2,667	1,800	1,467	270	833	1,467	7,333
	IR (%)	Control	75 (15/20)+	80 (28/35)	88 (29/33)	84 (16/19)	55 (22/40)	81 (26/32)*	64 (23/36)	59 (23/39)
		Starve	85 (17/20)	87 (34/39)	87 (33/38)	89 (17/19)	47 (18/38)	58 (22/38)	68 (25/37)	82 (28/34)*
Carcass	Median titre (PFU/mL)	Control	6,200	3,383	66,667	361,414	60	103	2,000	46,667
		Starve	73	5,017	69,167	193,333	0	163	1,067	100,000
	MER (%)	Control	20 (3/15)	50 (14/28)	66 (19/29)	75 (12/16)	5 (1/22)	15 (4/26)	61 (14/23)	74 (17/23)
		Starve	12 (2/17)	37 (14/38)	97 (32/33)*	94 (16/17)	0 (0/18)	9 (2/22)	84 (21/25)	89 (25/28)
	DR (%)	Control Starve	15 (3/20) 10 (2/20)	40 (14/35) 36 (14/39)	58 (19/33) 84 (32/38)*	63 (12/19) 84 (16/19)	3 (1/40) 0 (0/38)	13 (4/32) 5 (2/38)	39 (21/37) 57 (21/37)	44 (17/39) 74 (25/34)*

DPI: days post-infection

*: significantly higher titre (Mann-Whitney U-test) or infection/dissemination rate (Fisher’s exact test) than other treatment group

**+**: (number of tissues positive divided by the total number of tissues tested)

IR: Infection rate (number of infected midguts divided by the total number of midguts analysed) x 100

MER: Midgut escape rate (number of infected carcasses / number of infected midguts) x 100

DR: Dissemination rate (number of infected carcasses / total number of carcasses analysed) x 100

Quantification of virus by plaque assay revealed differences in midgut infection dynamics; at 5 dpi, Starve midguts had a significantly higher median titre of ZIKV relative to Control midguts (**Fig 3C** and **Table 3**; *p* = 0.02, Mann-Whitney U-test). From 7–9 dpi, Starve and Control midguts had similar ZIKV titres, however, by 11 dpi, midguts in Starve mosquitoes had a significantly lower median titre of virus, relative to Control midguts (*p* = 0.008, Mann-Whitney U-test). In the second experiment in which Control and Starve mosquitoes were challenged with a ZIKV-containing blood-meal, midguts were dissected at an earlier time point post-infection (3 dpi), and at 5, 7, and 9 dpi. Similar infection foci were observed *in situ* in Starve and Control midguts throughout the experiment (**S2 Fig**). Starve mosquitoes ingested (timepoint 0) significantly more ZIKV than Control mosquitoes (**Fig 3D**; *p* = 0.0006, Mann-Whitney U-test). Regardless, we observed increased midgut infections in Control mosquitoes at early timepoints when compared to Starve mosquitoes, as midguts of Control mosquitoes had a significantly higher titre of virus at 3 and 5 dpi, relative to Starve midguts (*p* = 0.02, *p* = 0.03, Mann Whitney U-test) (**Fig 3D**). Control and Starve mosquitoes had a similar midgut infection rate at 3 dpi (55% and 47%, respectively) (**Table 3**), however, at 5 dpi Control midgut infection rate peaked, with significantly more midguts infected (81%) relative to Starve midguts (58%) (*p* = 0.04, Fisher’s Exact Test). Following the peak infection rate, there was a decrease in the ZIKV infection rate of Control midguts and their viral titre. ZIKV titre in Control midguts dropped significantly from 5 dpi to 7 dpi (**Fig 3D** and **Table 3**; (*p* = 0.008, Mann-Whitney U-test). The midgut infection rate in Control mosquitoes dropped gradually from the peak at 5 dpi (81%) to 9 dpi (59%) (**Table 3**). In contrast, midgut infection rate in Starve mosquitoes increased gradually from 3 dpi (47%) to 7 dpi (68%), with significantly more Starve midguts infected relative to Control by 9 dpi (82%) (*p* = 0.04, Fisher’s Exact Test).

### Dissemination of ZIKV was elevated in Starve mosquitoes compared to Control

**Fig 4:**
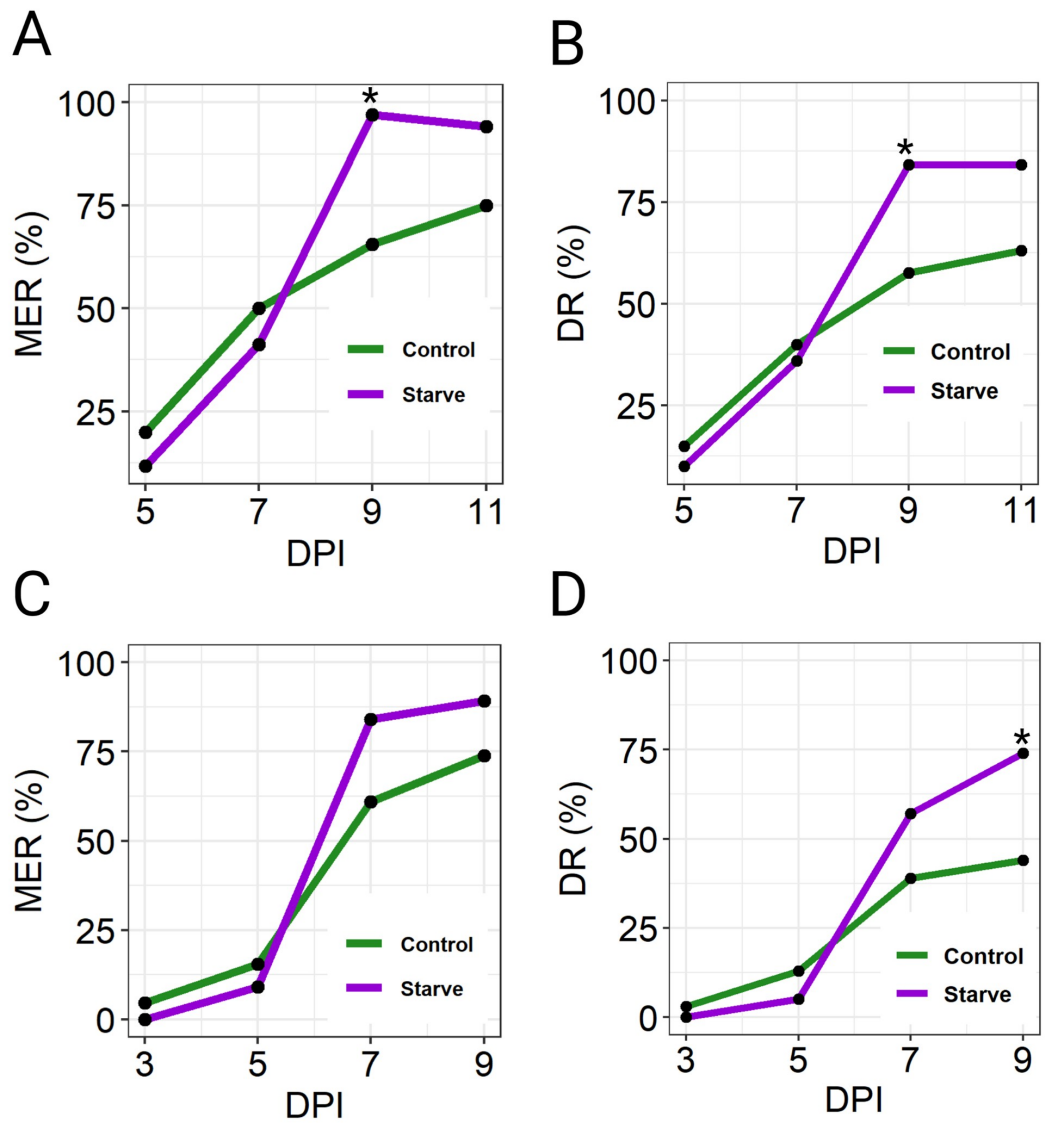
ZIKV I-44 midgut escape and dissemination rates in Control and Starve *Ae*. *aegypti* HWE following oral ingestion of virus in two independent experiments. The ZIKV titre in the blood-meal was 1.7x10^6^ plaque forming units (PFU) per mL. Virus was quantified in individual midguts and carcasses (n = 20–39 per group/timepoint) by plaque assay on Vero cells at 5, 7, 8, 9, and 11 days post-infection (dpi) in the initial experiment (**A**, **B**) and at 3, 5, 7 and 9 dpi in the second experiment (**C**, **D**). (**A**, **C**) ZIKV midgut escape rates (MER) for Starve and Control mosquitoes in experiment 1 (**A**) and experiment 2 (**C**), defined as the number of virus-positive carcasses divided by the number of virus-positive midguts over the time-course. (**B**, **D**) ZIKV dissemination rates (DR) for Starve and Control mosquitoes in experiment 1 (**B**) and experiment 2 (**D**), defined as the number of virus-positive carcasses divided by the total number of carcasses analysed. Count data (infected/non-infected tissues) were used for statistical analysis by Fisher’s Exact test; * = *p* < 0.05.

ZIKV prevalence and intensity of infection were also quantified in secondary mosquito tissues in absence of the midgut (hereby referred to as carcass for simplicity), representing virus infections following viral dissemination across the midgut BL. In both experiments, ZIKV titre and infection rates in the carcass increased in Control and Starve mosquitoes over the time-course (**Table 3**). The midgut escape rate (MER) of ZIKV in Control and Starve mosquitoes (number of infected carcasses divided by the number of infected midguts) was calculated, representing the midgut escape barrier. In the first virus challenge experiment, the MER increased in both treatment groups throughout the time-course; although similar MER were observed in Control and Starve mosquitoes at 5 dpi (20%, 12%, respectively) and 7 dpi (50%, 37%) (**Fig 4A** and **Table 3**). By 9 dpi, the MER in Starve mosquitoes (97%) increased significantly compared to Control mosquitoes (66%) (**Fig 4A**; *p* = 0.001, Fisher’s Exact test), with this trend continuing until 11 dpi (94%, 75%, respectively).

ZIKV dissemination rates from the midgut in the second experiment followed a similar trend as in the first experiment. Infection of carcasses at 3 dpi was minimal, with only 1/22 Control carcasses infected with ZIKV, while no detectable virus had disseminated in Starve carcasses (**Table 3**). ZIKV titre and infection rate of carcasses then increased in both treatment groups throughout the time-course (**Table 3**). The MER followed a similar pattern as observed in the first blood-meal challenge, with lower MER in Control and Starve mosquitoes at 3 dpi (5%, 0, respectively; **Fig 4C** and **Table 3**) and 5 dpi (15%, 9%), then a large increase in the MER at 7 dpi (61%, 84%) with this trend continuing until 9 dpi (74%, 89%).

The population dissemination rate (DR) of ZIKV in Starve and Control mosquitoes was calculated (number of virus-positive carcasses divided by the number of carcasses analysed) in both blood-meal challenge experiments, representing vector competence of both mosquito populations for ZIKV [27]. The DR increased in both mosquito populations throughout the time-course, however the DR within the Starve population was significantly higher than in the Control population at 9 dpi in the first experiment (84%, 58%; **Fig 4B** and **Table 3**; *p* = 0.02, Fisher’s Exact Test) and the second experiment (74%, 44%; **Fig 4D** and **Table 3**; *p* = 0.01, Fisher’s Exact Test).

### More ZIKV was released in saliva from Starve mosquitoes compared to the Control

Head tissue, carcasses and saliva samples were also collected from Starve and Control mosquitoes at 10 days following the challenge with a ZIKV-containing blood-meal in the previously mentioned second experiment. In this experiment, ZIKV intensity and prevalence of infection in head tissue were similar in both treatment groups (58% and 54% of Control and Starve heads infected, respectively) (**Table 4**). ZIKV prevalence in saliva samples was measured by assessing any occurrence of CPE in Vero cells on a daily basis, which had been inoculated with identical volumes (150μl/well) of the collected and diluted saliva samples at a defined timepoint. Control and Starve mosquitoes had a similar proportion of saliva samples infected, with 16% (3/19) and 15% (3/20) respectively (**Table 4**; **Experiment 2**). However, noticeable CPE (i.e., floating cells in proximity to a zone showing loosening of the cell mono-layer) occurred at 96 hours post-infection (hpi) for Starve saliva samples, compared to 144 hpi for Control samples. This indicates that Starve mosquitoes had a higher ZIKV concentration in their saliva than Control mosquitoes at 10 dpi.

**Table 4:** Median ZIKV titre (plaque forming units per mL) and infection rates in heads and carcasses of Starve and Control *Ae*. *aegypti* (HWE) at 10 and 14 days post-infection (dpi) and in saliva samples collected at 10 dpi.

Experiment 2				Experiment 3				
			10 DPI				10 DPI	14 DPI
Head	Median titre (PFU/mL)	Control	12667	Head	Median titre (PFU/mL)	Control	13000	66667
		Starve	12333			Starve	17333	93333*
	IR (%)	Control	58 (19/33)		IR (%)	Control	63 (19/30)	86 (18/21)
		Starve	54 (20/37)			Starve	72 (28/39)	96 (27/28)
Saliva	CPE	Control	96 h	Carcass	Median titre (PFU/mL)	Control	13000	66667
		Starve	144 h			Starve	17333	93333
	IR (%)	Control	16 (3/19)		IR (%)	Control	75 (30/40)	91 (21/23)
		Starve	15 (3/20)			Starve	98 (39/40)*	97 (28/29)

DPI: days post-infection with ZIKV-containing blood-meal

CPE: hours post-inoculation with saliva when cytopathic effects were observed on Vero cells

IR: Infection rate (Number of tissues infected/total tissues analysed)

*: significantly higher titre (Mann-Whitney U-test) or infection/dissemination rate (Fisher’s Exact test) than other treatment group

### ZIKV infection was elevated in tissue extremities in Starve mosquitoes compared to Control mosquitoes

Starve and Control mosquitoes were challenged with a ZIKV-containing blood-meal (4.5x10^5^ PFU/mL) in a third virus challenge experiment, and virus was quantified in the head and remaining carcass tissue at 10 and 14 dpi. The head tissue served as a proxy for dissemination to secondary organs such as salivary glands. Control and Starve mosquitoes ingested similar amounts of ZIKV, although the amount of imbibed virus was lower than in previous experiments as there was less virus provided in the blood-meal (170 and 167 PFU/mL, respectively; **S3 Fig**). In this experiment, the midgut was included within the carcass sample.

**Fig 5:**
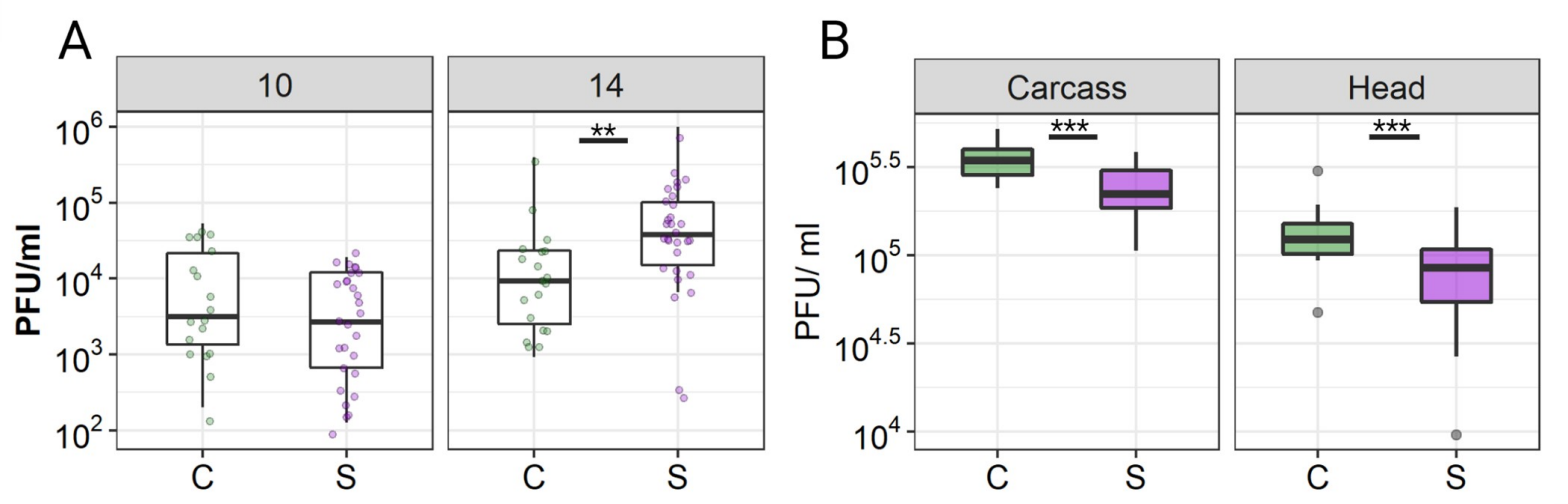
Dissemination and replication of ZIKV I-44 in tissue extremities of Control and Starve *Ae*. *aegypti* HWE following ingestion or intrathoracic injection of virus. **(A)** Starve and Control mosquitoes were fed a blood-meal containing 4.5x10^5^ PFU/mL ZIKV and virus was quantified in heads at 10 days post infection (dpi) (n = 19–28 per group/timepoint) and 14 dpi (n = 21–28 per group/timepoint) by plaque assay on Vero cells. Mann-Whitney U-test was used for statistical comparison; ** = *p* ≤ 0.01. Dots represent individual head tissues. (**B**) ZIKV was quantified in head tissue and carcasses of Starve and Control mosquitoes at 10 days post-intrathoracic injection of virus (around 140 PFU per mosquito; n = 20–21 per group). Outliers are shown as grey dots and were not included in the statistical analysis. T-test was used for statistical analysis (** = *p* ≤ 0.01, *** = *p* ≤ 0.001). Boxplots represent median titre, upper and lower quartiles with whiskers showing data extremities.

Significantly more Starve carcasses were infected at 10 dpi (98%) relative to Control (75%; *p* = 0.006, Fisher’s Exact Test) (**Table 4**; **Experiment 3**) whereas no significant difference in carcass infection rates was detected by 14 dpi between the two treatments. Median titre in carcasses increased in both treatment groups from 10 to 14 dpi (**Fig 5A** and **Table 4**). The head infection rate increased from 63% to 86% in Control mosquitoes from day 10 to 14, compared to 72% and 96% in Starve mosquitoes, respectively (**Table 4**). There was no difference in ZIKV titre in the heads between Control and Starve mosquitoes at 10 dpi, although by 14 dpi, there was a significantly higher ZIKV titre in Starve heads (*p* = 0.001, Mann-Whitney U-test) (**Fig 5A** and **Table 4**).

### ZIKV replication was inhibited in Starve mosquitoes compared to Control following intrathoracic injection

To assess whether Starve mosquitoes had an altered susceptibility to ZIKV in secondary tissues, we quantified ZIKV titre in Starve and Control mosquitoes following intrathoracic injection of ZIKV, thereby bypassing the midgut barriers. We injected 140 PFU ZIKV per female intrathoracically and quantified virus 10 days post-injection by plaque assay. Heads and carcasses were sampled and all tissues were infected with ZIKV in Control and Starve mosquitoes. Importantly, ZIKV titre was significantly lower in Starve carcasses (*p* = 0.0007, T-test) and heads (*p* = 0.009, T-test) relative to Control (**Fig 5B**) suggesting that ZIKV can replicate more efficiently in secondary tissues of the Control mosquitoes than in the Starve mosquitoes.

## Discussion

We exposed *Ae*. *aegypti* larvae to the stress of nutrient deprivation during rearing, resulting in small adults (Starve) that were compared to large adults (Control) in vector competence assays involving ZIKV. Starve mosquitoes had a higher vector competence for ZIKV than Control mosquitoes in three independent experiments. The midgut escape barrier was impaired leading to a higher MER in Starve mosquitoes in one experiment. The DR of ZIKV in the Starve mosquito population was significantly higher than in Control mosquitoes at 9 dpi in both midgut escape experiments. Given that ZIKV replication was inhibited in Starve mosquitoes following injection of virus, and more virus was present in head tissue and saliva of Starve mosquitoes compared to Control, we suggest that a higher quantity of virus was able to disseminate to secondary tissues across the diminished midgut escape barrier of the Starve mosquitoes.

Accordingly, the majority of the smaller Starve mosquitoes had a significantly thinner midgut BL compared to Control mosquitoes. However, at both timepoints sampled (5 and 7 dpi) there were outliers to this result, with one or two Starve midguts exhibiting a similar BL width as Control midguts. This may be due to larval competition during rearing that could be controlled for if each individual larva would be reared in a separate container for food supply. For example, within the Starve cohort, there may be individuals that ingested proportionally larger quantities of food than other larvae within the Starve group, which then developed a similar midgut BL width as some Control mosquitoes. As all Starve midgut BL were not impacted equally, this complicates the analysis of the effect a thinner BL may have on midgut escape. In addition, in all Control and Starve midguts visualized there was a wide variety of midgut BL width recorded, highlighting that the BL typically is not uniform in width throughout.

Despite this, Starve mosquitoes exhibited a higher midgut escape rate at 9 dpi in the first virus challenge experiment. Ultrastructural studies showed that ZIK virions were strongly accumulating at the midgut BL at 5 dpi, indicating that the virus can traverse the midgut BL after blood-meal digestion [16]. Therefore, it can be conceived that virions were traversing the midgut BL throughout the experiment, with the difference in carcass infection statistically observed at 9 dpi. Although we observed a similar trend in the second virus challenge experiment, this result was not statistically significant. A study investigating the vector competence of large and small *Culex tarsalis* for the flavivirus West Nile virus (WNV) showed in one experimental replicate a significantly higher infection rate in smaller mosquitoes than in large ones, however, this was not the case in every replicate performed [28]. Regardless, in both of our virus challenge experiments, Starve mosquito populations exhibited a higher vector competence for ZIKV relative to Control, as demonstrated by the DR. This suggests that epidemiologically, Starve mosquitoes may have a higher transmission potential for ZIKV than Control mosquitoes.

At 10 days post-intrathoracic injection of ZIKV, Starve mosquitoes contained less virus in head tissue and body than Control mosquitoes, indicating that replication in secondary tissues was impaired in the Starve mosquitoes. Despite this, we found higher titres of ZIKV in the head tissue of Starve mosquitoes 14 days post-infection with orally-acquired ZIKV, suggesting that a higher quantity of virions were eventually disseminating from midguts of the Starve mosquitoes than from those of the Controls. Accordingly, ZIKV has been previously shown to disseminate to the heads of *Ae*. *aegypti* at a higher rate in a dose-dependent manner [29]. Likewise, virus detection via amplification in Vero cells allows the conclusion that Starve mosquitoes released higher quantities of ZIKV along with saliva than Control females, although we cannot rule out that the barriers formed by the salivary glands were impaired in Starve mosquitoes.

In addition to the reduced titre following intrathoracic injection, ZIKV titre was also significantly lower in Starve midguts by 11 dpi in the first virus challenge experiment. These data suggest that in the smaller Starve mosquitoes, there were fewer cellular resources for ZIKV replication available at later timepoints during the infection process. Accordingly, smaller nutrient-deprived *Ae*. *aegypti* have been shown to have less protein, carbohydrate and lipid content than large mosquitoes [30]. Enveloped viruses such as ZIKV and dengue 1–4 viruses (DENV1-4) require components of intracellular membranes, including lipids, to facilitate their replication [31]. Consequently, lipids including fatty acyl, glycerophospholipid, and sphingolipid levels all increased specifically in the *Ae*. *aegypti* midgut in accordance with the replication kinetics of DENV2 [32].

We observed different midgut infection dynamics in the two virus challenge experiments and found that the amount of virus ingested from a blood-meal had no obvious effect on the subsequent midgut infection pattern. In the first virus challenge experiment, Starve and Control mosquitoes ingested similar amounts of ZIKV, yet by 5 dpi midguts in Starve mosquitoes exhibited a significantly higher ZIKV titre, which could be an indication for a diminished midgut infection barrier. At this timepoint we also observed several Starve midguts exhibiting a widespread punctate infection pattern based on ZIKV antigen detection *in situ*, in comparison to zonal antigen patches detected in the Control midguts. However, in the second ZIKV challenge experiment, Starve mosquitoes ingested significantly more virus, while Control midguts had a higher ZIKV titre at 3 and 5 dpi, indicating that indeed there was an effective midgut infection barrier present in the latter. Currently, we cannot explain the observation of a higher ZIKV titre in Starve midguts at 5 dpi in the first challenge experiment, leaving room for the speculation that an altered nutrient status could have affected antiviral immune responses or other essential pathways in the mosquito midgut, leading to the variation in midgut intensity of infection and infection prevalence at specific days. As shown by other authors, small *Ae*. *aegypti* females resulting from sub-optimal nutrient supply showed altered expression of many transcripts related to metabolism, immunity, apoptosis and reproduction, in comparison to large, well-fed individuals [33]. Following the ingestion of a blood-meal, overall metabolism was increased in small *Ae*. *aegypti* mosquitoes in comparison to large mosquitoes while fecundity was decreased in the former suggesting that in small size mosquitoes, the blood-meal was predominantly processed for nutrient supply rather than diverted for egg development [33]. In addition, the midgut microbiome in adult mosquitoes has been shown to be altered by restricting the larval diet [34]. This, in turn, can impact midgut infection of arboviruses; for instance, a microbial metalloprotease secreted by *Serratia marcescens* increased the susceptibility of the *Ae*. *aegypti* midgut to DENV2 infection [35]. In the second viral challenge experiment, the midgut infection rate of the Control peaked at 5 dpi, followed by gradual decrease in infection until 9 dpi causing Starve mosquitoes to exhibit a comparatively higher midgut infection rate with ZIKV at 9 dpi. It is unclear why these differences in midgut infection occurred, yet it highlights the variability that can occur in *Ae*. *aegypti* vector competence studies when repeatedly performed under similar conditions. Despite the differences in midgut infection rate, the midgut dissemination rates were similar among the Control mosquitoes of both experiments.

Previous studies investigating the vector competence for arboviruses in small and large *Aedes* mosquitoes have shown varied results as well, as demonstrated by the following examples. Large *Ae*. *aegypti* females had higher infection rates with DENV2 (10.7%) compared to small mosquitoes (5.7%) at 14 days post-challenge with a DENV2-containing blood-meal [36]. On the other hand, another study found small and large *Ae*. *aegypti* produced by overcrowding with its own species or in competition with *Ae*. *albopictus* exhibited no differences in vector competence for DENV2 [37]. However, smaller *Ae*. *albopictus* reared under the same conditions had at least 60% more DENV2 dissemination compared to those reared optimally. Larger *Ae*. *aegypti* had higher infection rates with alphavirus Ross River Virus (RRV) in the whole body than small mosquitoes [38]. Another study that investigated infection of RRV in the body, head, and salivary glands of *Ae*. *vigilax* found no difference in infection rate and viral titre between small, medium, and large mosquitoes over a time-course [39]. Larger *Ae*. *albopictus* produced by rearing larvae at cooler temperatures had significantly higher infection and population dissemination rates of chikungunya virus (*Togaviridae*; *Alphavirus*) than small mosquitoes reared at warmer temperatures [27]. Small and large *Cx*. *annulirostris* showed no difference in their vector competence for the flavivirus Murray Valley encephalitis virus at 10 dpi [40]. Thus, a direct comparison of studies involving different mosquito strain-virus strain combinations faces its limitations, as overall vector competence may intrinsically differ for each mosquito strain (species)–virus strain (species) pairing, and different stressors may impact the mosquito in various ways.

## Conclusions

This study highlights the impact nutritional stress during larval development can have on the dynamics of ZIKV infection in *Ae*. *aegypti* females and their transmission potential of the virus. Small *Ae*. *aegypti* adults that were deprived of food as larvae had a thinner midgut BL, the physical evidence for a midgut escape barrier, compared to optimally reared Control mosquitoes. Small *Ae*. *aegypti* had a higher midgut escape rate in one experiment and more virus reaching tissue extremities including saliva, despite virus replication inhibited in small *Ae*. *aegypti* as shown by intrathoracic injection of ZIKV. These data suggest that an impaired midgut escape barrier was contributing to the higher dissemination rates of virus. Thus, variation in larva nutrition is potentially a source for the variation of female vector competence for ZIKV.

## Supporting information

S1 Table Feeding regimen to produce large (Control) and small (Starve) *Ae*. *aegypti* HWE. Larvae were hatched and given optimal food (Control) or restricted food quantities (Starve) to produce small and large adults. Control larvae were hatched two days after Starve larvae to account for delayed pupation times in the latter.
(TIF)

S1 Fig Measurements of wings from large (Control) and small (Starve) *Ae*. *aegypti* HWE. (**A**) Adult wings were dissected and mounted onto a microscope slide with double-sided Scotch tape for visualization using a Leica ICC50 Compound Microscope equipped with camera. Area measured is indicated by a white arrow. ImageJ was used to measure the wing lengths (mm) from three independent replicates. (**B**) Starve mosquitoes had significantly smaller wings than Control in three independent replicates. Boxplots represent data from 6–10 mosquitoes per experiment with the median, upper and lower extremities shown. Statistical analysis was based on Mann-Whitney U-test, *** = *p* < 0.0001.
(TIF)

S2 Fig Midgut infection foci of ZIKV I-44 in Starve and Control *Ae*. *aegypti* HWE as shown by immunofluorescence (IFA). Detection of ZIKV antigen in the second experiment investigating midgut infection following ingestion of a blood-meal containing ZIKV I-44. Six midguts were analysed per time-point at 3, 5, 7, and 9 days post-infection. Fixed midguts were incubated with the flavivirus-specific 4G2 primary mouse monoclonal antibody and secondary anti-mouse Alexa Fluor (AF) 594 labeled monoclonal antibody (red). Actin filaments were stained using Alexa Fluor (AF) Phalloidin 488 (green); nuclei were stained using DAPI (blue). Mock samples show non-infected midguts which underwent the same staining procedure as the infected midguts. Images are shown at 10x magnification.
(TIF)

S3 Fig Amount of ZIKV I-44 ingested by Starve and Control *Ae*. *aegypti* HWE from a blood-meal in experiment 3. ZIKV was quantified in whole bodies of Control and Starve mosquitoes immediately after ingestion of a blood-meal (timepoint 0). n = 5–6 mosquitoes. Statistical analysis was based on T-test (*p* = 0.90).
(TIF)
