# Supplementary figures and images for: Starvation at the larval stage increases the vector competence of Aedes aegypti females for Zika virus
Source: PLoS Negl Trop Dis. 2021 Nov 29;15(11):e0010003. doi: 10.1371/journal.pntd.0010003 (PMC8659361; doi:10.1371/journal.pntd.0010003)

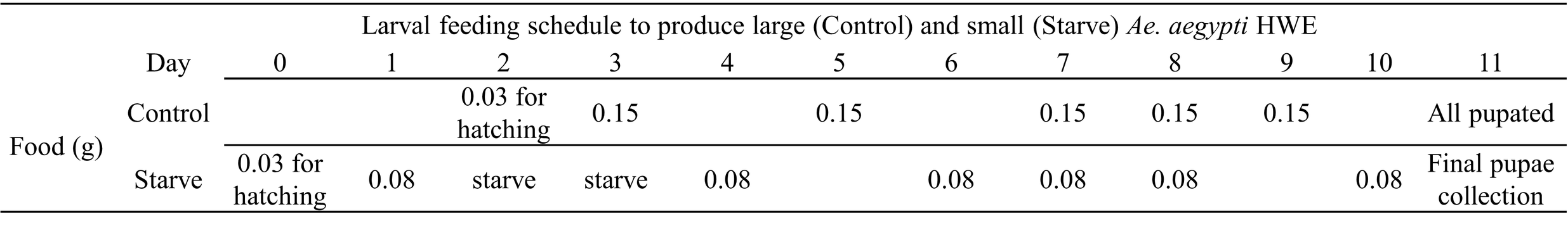

Supplement: S1 Table — Larvae were hatched and given optimal food (Control) or restricted food quantities (Starve) to produce small and large adults. Control larvae were hatched two days after Starve larvae to account for delayed pupation times in the latter. (TIF) [file pntd.0010003.s001.tif]

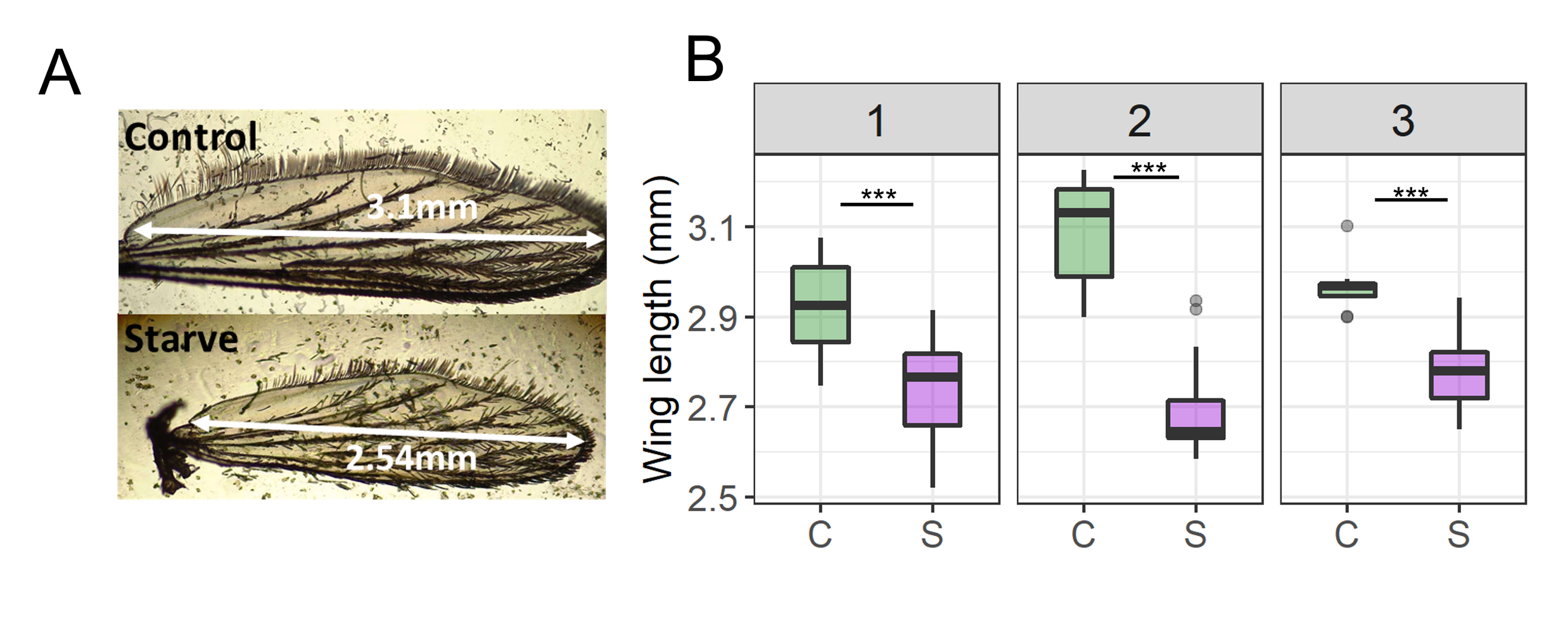

Supplement: S1 Fig — (A) Adult wings were dissected and mounted onto a microscope slide with double-sided Scotch tape for visualization using a Leica ICC50 Compound Microscope equipped with camera. Area measured is indicated by a white arrow. ImageJ was used to measure the wing lengths (mm) from three independent replicates. (B) Starve mosquitoes had significantly smaller wings than Control in three independent replicates. Boxplots represent data from 6–10 mosquitoes per experiment with the median, upper and lower extremities shown. Statistical analysis was based on Mann-Whitney U-test, *** = p < 0.0001. (TIF) [file pntd.0010003.s002.tif]

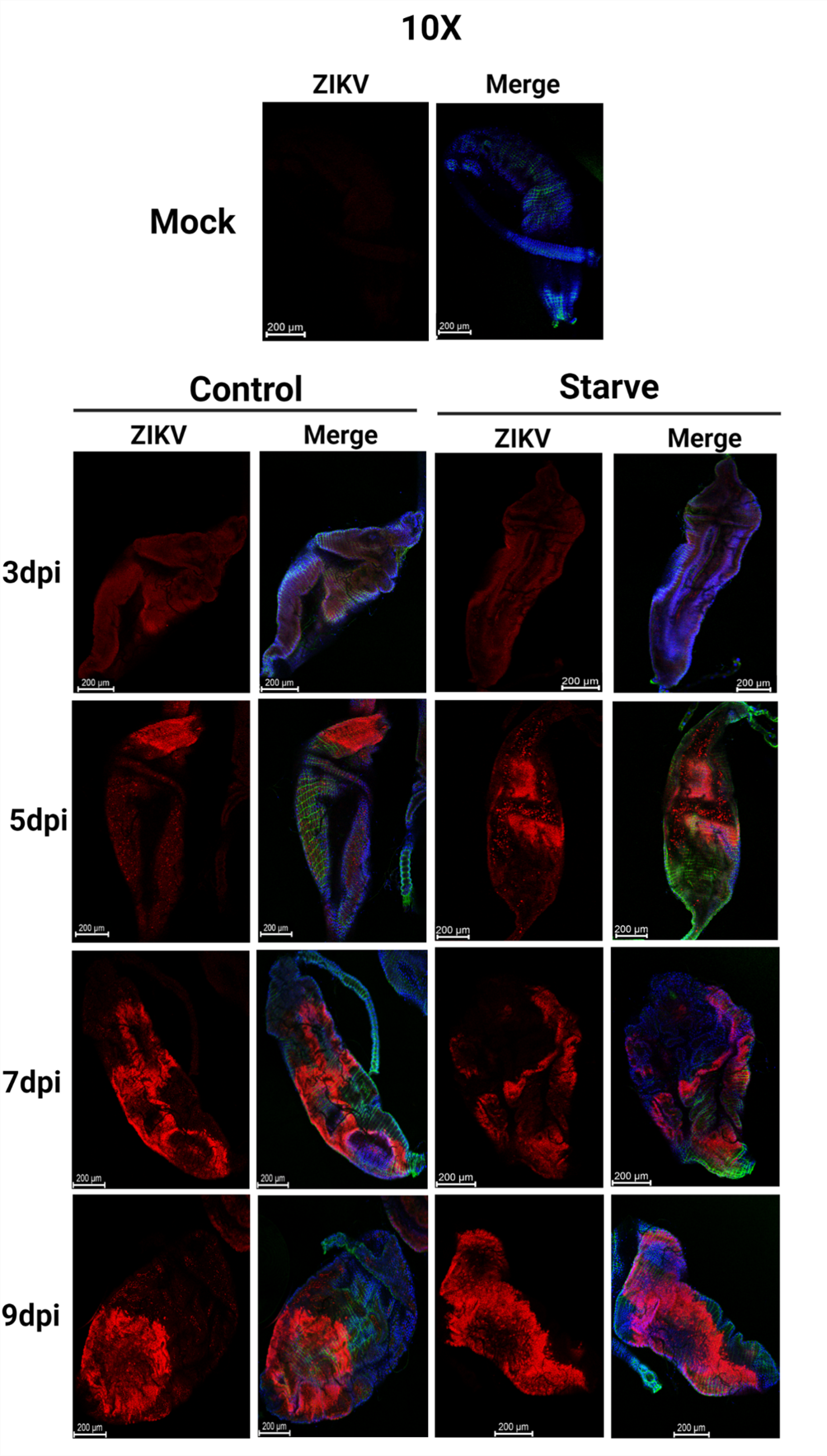

Supplement: S2 Fig — Detection of ZIKV antigen in the second experiment investigating midgut infection following ingestion of a blood-meal containing ZIKV I-44. Six midguts were analysed per time-point at 3, 5, 7, and 9 days post-infection. Fixed midguts were incubated with the flavivirus-specific 4G2 primary mouse monoclonal antibody and secondary anti-mouse Alexa Fluor (AF) 594 labeled monoclonal antibody (red). Actin filaments were stained using Alexa Fluor (AF) Phalloidin 488 (green); nuclei were stained using DAPI (blue). Mock samples show non-infected midguts which underwent the same staining procedure as the infected midguts. Images are shown at 10x magnification. (TIF) [file pntd.0010003.s003.tif]

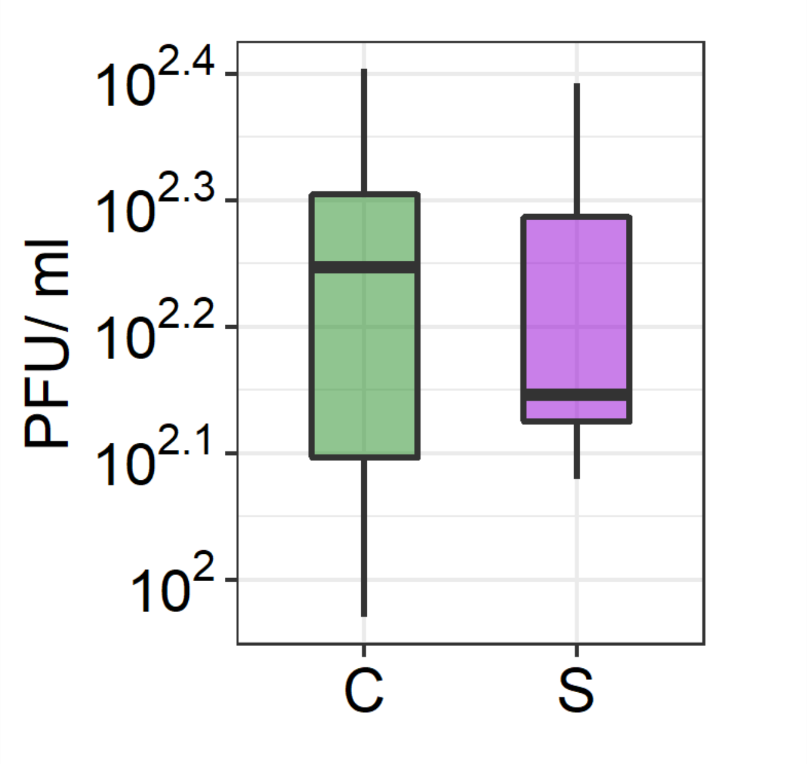

Supplement: S3 Fig — ZIKV was quantified in whole bodies of Control and Starve mosquitoes immediately after ingestion of a blood-meal (timepoint 0). n = 5–6 mosquitoes. Statistical analysis was based on T-test (p = 0.90). (TIF) [file pntd.0010003.s004.tif]
